# Cerebral Near Infrared Spectroscopy Monitoring in Term Infants With Hypoxic Ischemic Encephalopathy—A Systematic Review

**DOI:** 10.3389/fneur.2020.00393

**Published:** 2020-05-27

**Authors:** Subhabrata Mitra, Gemma Bale, Judith Meek, Ilias Tachtsidis, Nicola J. Robertson

**Affiliations:** ^1^Neonatology, Institute for Women's Health, University College London, London, United Kingdom; ^2^Medical Physics and Biomedical Engineering, University College London, London, United Kingdom

**Keywords:** neonate, hypoxic ischemic encephalopathy, oxygenation, metabolism, near infrared spectroscopy

## Abstract

**Background:** Neonatal hypoxic ischemic encephalopathy (HIE) remains a significant cause of mortality and morbidity worldwide. Cerebral near infrared spectroscopy (NIRS) can provide cot side continuous information about changes in brain hemodynamics, oxygenation and metabolism in real time.

**Objective:** To perform a systematic review of cerebral NIRS monitoring in term and near-term infants with HIE.

**Search Methods:** A systematic search was performed in Ovid EMBASE and Medline database from inception to November 2019. The search combined three broad categories: measurement (NIRS monitoring), disease condition [hypoxic ischemic encephalopathy (HIE)] and subject category (newborn infants) using a stepwise approach as per PRISMA guidance.

**Selection Criteria:** Only human studies published in English were included.

**Data Collection and Analysis:** Two authors independently selected, assessed the quality, and extracted data from the studies for this review.

**Results:** Forty-seven studies on term and near-term infants following HIE were identified. Most studies measured multi-distance NIRS based cerebral tissue saturation using monitors that are referred to as cerebral oximeters. Thirty-nine studies were published since 2010; eight studies were published before this. Fifteen studies reviewed the neurodevelopmental outcome in relation to NIRS findings. No randomized study was identified.

**Conclusion:** Commercial NIRS cerebral oximeters can provide important information regarding changes in cerebral oxygenation and hemodynamics following HIE and can be particularly helpful when used in combination with other neuromonitoring tools. Optical measurements of brain metabolism using broadband NIRS and cerebral blood flow using diffuse correlation spectroscopy add additional pathophysiological information. Further randomized clinical trials and large observational studies are necessary with proper study design to assess the utility of NIRS in predicting neurodevelopmental outcome and guiding therapeutic interventions.

## Introduction

The current practice of therapeutic hypothermia (TH) has reduced the disability rates and the severity spectrum of cerebral palsy in newborn infants with hypoxic ischaemic encephalopathy (HIE). Infants with HIE are routinely monitored in the neonatal intensive care unit (NICU) with electroencephalography (EEG) or amplitude integrated EEG (aEEG) and cranial ultrasound (CrUSS) during TH to detect seizures, investigate the severity of the injury and inform prognosis. Brain magnetic resonance imaging (MRI) and spectroscopy (MRS) are gold standard tools for prognostication of injury and are optimally performed after completion of TH. However, these current technologies do not offer all the necessary physiological information that is needed for a continuous assessment of the changes in the newborn brain.

Following perinatal hypoxia-ischaemia, significant cerebral haemodynamic and metabolic derangements evolve over time (1–3). This evolution is associated with changes in the brain energy state and underlying neurochemical and neurotoxic state. Following acute hypoxia ischaemia (HI), brain high energy metabolites decrease with a reduction in CBF. Oxidative metabolism appears to recover after resuscitation along with improvement in brain perfusion but a period of hypoperfusion persists in the latent phase (up to 24 h following injury). Without any intervention, mitochondrial failure and cell death start in the secondary phase (persisting for days after birth) together with a state of hyperperfusion. Over the next few weeks and months, cerebral hemodynamic and metabolic abnormalities gradually normalize despite pathological processes persisting in this tertiary phase of injury. The degree of deranged cerebral oxidative metabolism following HIE can be identified using magnetic resonance spectroscopy (MRS) (4, 5).

Cerebral NIRS has several advantages as a neuromonitoring tool in the neonatal intensive care unit (NICU) and can be combined with aEEG/EEG monitoring. Its application is easy and quick. The monitoring can be continuous over a long period of time. NIRS can continuously monitor CBF, oxygenation, and metabolism at the cot side from the early stages after birth, with the potential to provide information on the severity of the evolving injury and outcome. NIRS uses the relative transparency of biological tissue in the near infrared (NIR) region of light (700–1,000 nm). In comparison to adults and older children, the thinner skin and skull thickness in newborn infants allows a better depth penetration of brain tissue and make the technique an ideal neuromonitoring tool for newborn infants. Hemoglobin is one of the compounds (chromophores) in the human body that absorbs light. The absorption spectra of oxygenated and de-oxygenated hemoglobin (HbO_2_ and HHb) are different in the near-infrared region, allowing changes in concentration to be individually monitored using NIRS. Total hemoglobin (HbT = HbO_2_ + HHb) and hemoglobin difference (HbD = HbO_2_-HHb) are derived parameters and have been used to represent changes in cerebral blood volume (CBV) and cerebral oxygenation, respectively. Most commercially available NIRS systems measure cerebral oxygenation or tissue saturation (StO_2_, rScO_2_, TOI, rSO_2_), which is the percentage ratio of HbO_2_ to HbT (HbO_2_/HbT); these systems are often referred to as brain oximeters, with different manufactures implementing different NIRS techniques to derive brain tissue saturation (6–9). We will use the term cerebral oxygenation in this review and this measurement will be discussed in further detail later under “NIRS devices and methodology.” In healthy term infants, irrespective of the mode of delivery, cerebral oxygenation is lowest at birth (between 40 and 56%) (6–10) and gradually increases to reach ~78% (±7.9%) in the first 24 h (11). It stabilizes over the next few weeks between 55 and 85% (12–14). Using the combined measurement of cerebral oxygenation and peripheral arterial oxygen saturation one can estimate fractional tissue oxygen extraction (FTOE = SaO_2_ – cerebral oxygenation/SaO_2_). It represents the balance between oxygen delivery and oxygen consumption, a proxy marker of cerebral metabolism (15). An increase in FTOE indicates an increased extraction of oxygen by brain tissue, suggesting a higher oxygen consumption in relation to oxygen delivery. A decrease of FTOE, on the other hand suggests reduced use of oxygen by brain tissue in relation to supply. Neither cerebral oxygenation nor FTOE accurately reflects the cerebral metabolic rate of oxygen consumption (CMRO_2_).

Beyond the NIRS measurements of hemoglobin oxygenation, another NIR chromophore is cytochrome c oxidase (CCO). It is the terminal electron acceptor in the electron transport chain (ETC) and is responsible for more than 90% of ATP production, thus providing important information related to the changes in mitochondrial oxidative metabolism. The absorption spectra of the CCO will depend on its redox state, which in turn will depend on oxygen and energetic substrate availability. The NIRS measurements of CCO attracted a great deal of attention in the 1980's (16–19) but accurate measurement of CCO proved challenging due to its low *in vivo* concentration. However, recent developments in optical methods have enabled this measurement in the NICU, as will be discussed later under “NIRS devices and methodology.”

We performed a systematic review of NIRS measurements in term or near-term newborn infants with HIE. Although several NIRS reviews in the preterm population have been published (6, 20), there is no comprehensive review of NIRS in term infants with HIE. The aim of this work is to review the potential benefits of cerebral NIRS monitoring in newborn infants with HIE and the utility of different NIRS variables to prognosticate outcome (short term or long term). We present a review of different optical measurements: how these indices evolve over time and their relationship with outcome, monitoring of cerebral autoregulation using NIRS and the use of NIRS together with other neuromonitoring tools. A brief description of the basic methodology and technology used for cerebral NIRS is presented along with emerging technologies in this area.

## Methods

A stepwise approach was taken to identify articles from databases following the guidance from the Preferred Items for Systematic Reviews and Meta-analysis (PRISMA) statement (21).

## Search Strategy

Published articles were identified using a systematic search of Medline database and Ovid EMBASE from inception to November 2019. Articles were filtered with publications in English only. The search focused on publications related to cerebral oxygenation, perfusion and metabolism using single or multiple site NIRS in infants with HIE. The retrieved articles were further examined for any other relevant published reports. Websites of manufacturers of NIRS monitors were also screened during the search to capture any articles that might have been overlooked.

The search combined three broad categories: measurement (NIRS monitoring), disease condition [hypoxic ischemic encephalopathy (HIE)] and subject category (newborn infants). Search term included: brain metabolism, brain function, tissue metabolism, cerebral metabolism or oxygenation or hemodynamic or blood flow or volume, near infrared, near infrared spectroscopy, brain hypoxia or anoxia, perinatal hypoxia or ischemia, asphyxia neonatorum, brain ischemia, brain injury or damage, encephalopathy, neonatal encephalopathy, newborn(s), newborn babies, infant(s) and neonate, neonates and neonatal.

The search strategies for the review is presented in Table 1.

**Table 1:** Search strategy for the systamtic review on EMBASE.

**Search strategy used for systematic review:**
1. (“exp brain metabolism”) or (“brain function”) or (“tissue metabolism”) or ([cerebr^*^ adj3 (metabol^*^ or oxygenation or hemodynamic^*^ or “blood flow” or volume^*^)].mp. [mp=title, abstract, heading word, drug trade name, original title, device manufacturer, drug manufacturer, device trade name, keyword, floating subheading word, candidate term word]) or (“near infrared spectroscopy”) or ((near infrared adj3 spectroscop^*^).mp. [mp=title, abstract, heading word, drug trade name, original title, device manufacturer, drug manufacturer, device trade name, keyword, floating subheading word, candidate term word]). (Result: 169743)
2. (“brain hypoxia”) or (“exp brain ischemia”) or [((cerebr^*^ or brain^*^) adj3 (“brain damage” or encephalopath^*^)].mp. [mp=title, abstract, heading word, drug trade name, original title, device manufacturer, drug manufacturer, device trade name, keyword, floating subheading word, candidate term word]) or [((hypoxi^*^ or anoxi^*^) adj3 (“brain damage^*^” or “brain injur^*^” or encephalopath^*^)].mp. [mp=title, abstract, heading word, drug trade name, original title, device manufacturer, drug manufacturer, device trade name, keyword, floating subheading word, candidate term word]) or (“perinatal asphyxia”) or [(asphyxia^*^ adj3 (newborn or baby or babies or infan^*^ or neonat^*^)].mp. [mp=title, abstract, heading word, drug trade name, original title, device manufacturer, drug manufacturer, device trade name, keyword, floating subheading word, candidate term word]) or ((encephalopath^*^ adj3 neonat^*^).mp. [mp=title, abstract, heading word, drug trade name, original title, device manufacturer, drug manufacturer, device trade name, keyword, floating subheading word, candidate term word]). (Result: 228291)
3. (‘newborn’) or ((newborn or baby or babies or infan^*^ or neonat^*^).mp. [mp=title, abstract, heading word, drug trade name, original title, device manufacturer, drug manufacturer, device trade name, keyword, floating subheading word, candidate term word]). (Result: 1475873)
4.1 and 2 and 3. (Result: 1671)

## Study Selection

Publications were included in the review if they presented original data discussing the use of NIRS in newborn term or near-term infants with HIE. The articles identified from the databases were screened for duplicity and were then evaluated using the publication title and abstracts. Full texts were examined where uncertainty was noted at this stage. Articles with preclinical studies and abstract-only publications (where a full-length article was not published in a peer-reviewed journal) were excluded. Studies on human infants were included for the final analysis. Full texts were assessed for the remaining articles and publications were excluded if they did not present original data in the term newborn population (review articles, commentaries, and studies in preterm population). Studies included for the final review were then assessed for data collection.

## Results

**Figure 1:**
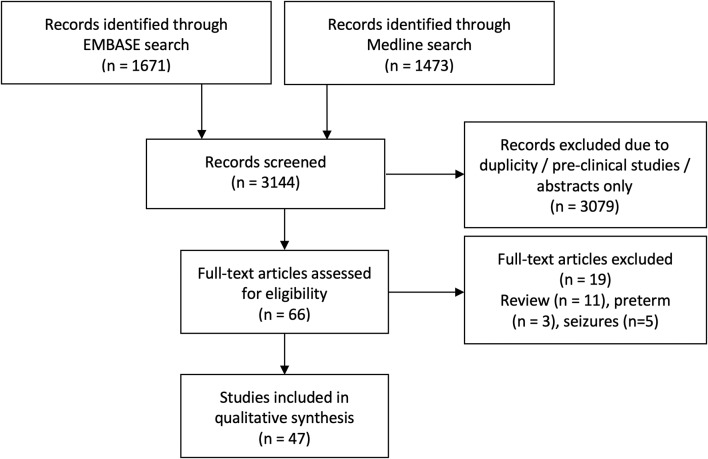
PRISMA flow chart for the systamatic review.

**Figure 2:**
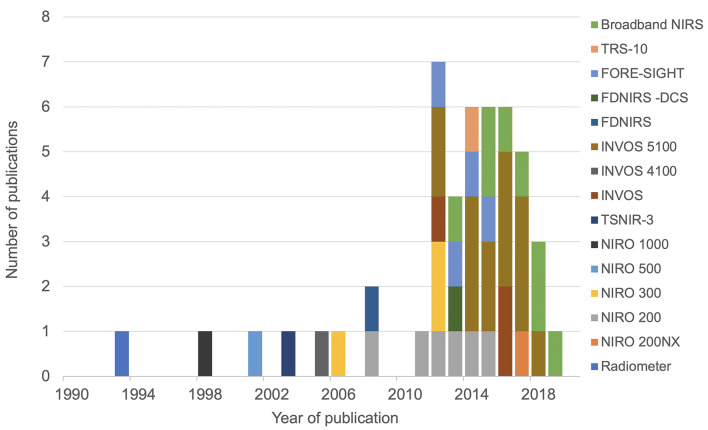
Different NIRS devices used in the studies with their year of publication.

The initial search identified 3,144 articles. After excluding duplicate articles, preclinical studies and abstract only publications, 66 articles were identified, all of which discussed NIRS measurement in human newborn infants after HIE. Full texts of these articles were reviewed, and a further 14 articles were excluded (review papers:11, and studies in preterm population: 3), leading to final inclusion of 52 original research studies on the term and near-term infants following HIE. Five studies presented changes in NIRS variables specifically during seizures following HIE and were excluded from this review leaving 47 studies for the final review. The PRISMA chart detailing the searching and inclusion of the articles for the review is presented in Figure 1. Basic characteristics and brief details of the studies are presented in Table 2. Thirty-nine studies have been published since 2010, compared to eight studies before 2010. Most of the studies (thirty-five) investigated the changes in cerebral oxygenation. Other optical indices were FTOE, CBF, CMRO_2_, HbD, HbT, oxCCO, markers of haemodynamic reactivity and metabolic reactivity. Fifteen different devices were used in these studies and eleven of them were commercially available (Figure 2). Eight recent studies used broadband NIRS (BNIRS) to investigate changes in cerebral metabolism using direct measurement of changes in the oxidation state of cytochrome c oxidase (oxCCO). Fifteen studies presented MRI or MRS evidence of injury as short term outcome for comparing with NIRS makers while an equivalent number of studies presented neurodevelopmental follow up data. Eight studies used combined neuromonitoring using NIRS and aEEG/EEG.

**Table 2:** Study characteristics.

**First author, Year (Ref.)**	**Study design**	**Gestation (weeks)**	**No of subjects**	**Study aim**	**NIRS device, sensor**	**Optode placement on head**	**Duration of study**	**Result**
Ancora et al. (22)	Observational	39	1	To evaluate the time course of aEEG and NIRS data before, during and after cool cap treatment	NIRO 200	Forehead		Early significant increase in THI and TOI before TH. TOI improved with TH and remained stable during the rewarming period
Ancora et al. (23)	Observational	≥36	12	To evaluate the prognostic value NIRS data in asphyxiated cooled infants	NIRO 200	Forehead	72 h	Mean TOI at 12h of life is significantly higher in infants who develop a poor neurological outcome than in those with normal outcome
Arriaga-Redondo et al. (24)	Observational	≥36	23	To assess the variability of cerebral tissue oxygenation over time in infants with HIE	Invos 5100	Forehead	100 h	rScO_2_ values >90% and a lack of variability over time in infants with HIE during cooling were associated with poor outcome
Bale et al. (25)	Observational	≥38	6	Feasibility study to assess the potential of cytochrome c oxidase monitoring in NE	Broadband NIRS	Frontal bilateral	Up to 5 days	Mean values for HbD and oxCCO consistently decreased during desaturation and HbT increased.
Bale et al. (26)	Prospective observational	>35	11	To investigate the dynamic changes in cerebral metabolism in response to systemic changes, as a marker of injury	Broadband NIRS	Frontal	3 h on day 3 of TH	Strong relationship between oxCCO and systemic variables during TH on day 3 indicated severe injury following HI
Bale et al. (27)	Prospective observational	>36	50	To determine whether broadband NIRS can distinguish injury severity in HIE in the first 4 days after birth	Broadband NIRS	Frontal	Up to day 4	A strong relationship between cerebral metabolism [broadband NIRS-measured cytochrome-c-oxidase (CCO)] and cerebral oxygenation was associated with unfavorable outcome during spontaneous desaturation episodes during TH.
Bale et al. (28)	Observational	>36	11	To use changes in cerebral oxygenation and peripheral oxygen saturation during spontaneous desaturation for determination of CBF	Broadband NIRS	Frontal	Up to day 4	Infants with severe HIE had significantly lower CBF compared with infants with moderate HIE on the day of birth
Burton et al. (29)	Observational	≥35	19	To assess the relationship between autoregulation during TH and neurodevelopmental outcomes at 2 years of age	INVOS 5100	Forehead	84 h	Children with developmental impairments at 2 years, had higher MAP_OPT_ values, spent more time with MABP below MAP_OPT_, and had greater MABP deviation below MAP_OPT_ during rewarming. Greater MABP deviation above MAP_OPT_ during rewarming was associated with less disability and higher cognitive scores
Campbell et al. (30)	Observational	>36	27	Whether infants with autonomic dysfunction after HIE have aberrant physiological responses to care events	NIRO-200NX	Right and left frontotemporal	2.58–8 h	Infants with depressed heart rate (HR) variability had different physiological responses [post event changes in cerebral blood flow (HbD) and cerebral blood volume (HbT)] compared to infants with intact HR variability
Chalak et al. (31)	Observational	≥36	10	To develop an approach to assess cerebral hemodynamics across multiple time scales during first 72 h of life	INVOS 4100–5100	left frontoparietal	72 h	multiple-timescale correlations between oscillations in MAP and SctO_2_ in the first 72 hrs, indicating impairment of cerebral hemodynamics
Chalak et al. (32)	Observational	≥36	10	To quantify neurovascular coupling (NVC) using wavelet analysis of the dynamic coherence between aEEG and SctO_2_ in NE	INVOS 4100–5100	Bilateral parietal area	60 ± 6 h	High coherence, intact NVC between the oscillations of SctO_2_ and aEEG in the frequency range of 0.00025–0.001 Hz in the non-encephalopathic newborns. NVC coherence was significantly decreased in encephalopathic newborns who were cooled vs. non- encephalopathic controls and was significantly lower in those with abnormal 2 year outcomes relative to those with normal outcomes
Chen et al. (33)	Observational	>35.7	44	To evaluate the evoked CBO response to neuronal activation in newborns with HIE and compare with the response in healthy infants	NIRO 500	Forehead	Between day 1–3	Infants with HIE have decreased rCBF in the frontal lobes during auditory stimulation, (decrease of HbO_2_ and HbT) compared to normal infants
Chock et al. (34)	Retrospective chart review	≥36	38	To review cerebral and renal tissue saturation during TH	INVOS 5100C	Lateral forehead	110 h	Renal tissue saturation was lower than cerebral tissue saturation during TH
Dehaes et al. (35)	Observational	≥36	27	To assess cerebral hemodynamics and oxygen metabolism during and after TH	Hybrid FDNIRS–DCS system	Left, middle, and right frontal	10–16 sec 3 times/ location during TH, rewarming, and post-TH	CMRO_2i_ and CBF lower in neonates with HIE during TH compared with post –TH and controls
Forman et al. (36)	Prospective observational	>35	20	To assess the feasibility and reliability cerebral perfusion monitoring in NE	INVOS, neonatal sensors	Center of the forehead	84 h	SctO_2_ increased over first 30 h of TH and stayed high for the remainder of the study
Gagnon and Wintermark (37)	Case series	>38	3	To examine the impact of PPHN on cerebral oxygenation in infants on TH after HIE	FORE-SIGHT	Forehead (bilateral)	86 h	Periods of pulmonary hypertensive crisis were associated with significant drop in cerebral saturation, indicating that PPHN can independently cause further injury
Goeral et al. (38)	Prospective observational	>36	32	To assess the predictive values of aEEG and NIRS parameters and the respective cut-off values regarding short-term outcomes in HIE	INVOS 5100C	Frontoparietal	102 h	No significant differences in NIRS values were observed between groups (normal and abnormal MRI). Combined score of BP, aEEG and NIRS increased the accuracy of early outcome prediction
Govindan et al. (39)	Observational	n.r.	4	To identify the efficacy of a modified approach to quantify the pressure passivity	NIRO 200	Bilateral fronto-temporal areas	n.r.	A modified coherence estimation approach over every 30 s epochs identified better the association between HbD and MABP (pressure passivity index).
Govindan et al. (40)	Observational	≥38	4	To review the efficacy of a novel method to quantify neuro-vascular coupling (NVC) using NIRS and EEG	NIRO 200	Bilateral fronto-temporal areas	n.r.	Two infants who survived, revealed the emergence of NVC during TH. Other 2 infants who did not survive, lacked this feature.
Grant et al. (41)	Observational	≥33	43	Whether StO2, CBV, and rCMRO_2_ have the potential to distinguish between neonates with brain injury (HIE and other etiologies) and healthy controls	FDNIRS	5 ± 3 locations. Primary location –forehead, also temporal and parietal	n.r.	No significant difference in StO_2_ between brain-injured and normal neonates. However, CBV and estimates of rCMRO_2_ were significantly increased in the brain injured group compared with all other clinical groups
Gucuyener et al. (42)	Observational	≥36	8	Investigate the correlations between aEEG and NIRS monitoring and outcome following HIE	NIRO 200	Parietal	30 min each before cooling, at 34°C during TH and after rewarming	Detection of context-sensitive changes in TOI and FTOE can be helpful especially while monitoring the effects of a therapy, in conjunction with other cerebral trend monitors
Howlett et al. (43)	Observational	>37	24	To describe the relationship between autoregulation during TH and brain injury on MRI after HIE	INVOS, Neonatal sensor	Forehead	84 h	Optimal MABP identified using HVx (running correlation between HbT and MAP). Infants with evidence of brain injury on MRI spent longer time below MAP_OPT_ during rewarming than neonates with no or mild injury. Neonates with moderate/severe injury on MRI had greater MAP deviation below MAP_OPT_ during rewarming than neonates without injury
Huang et al. (44)	Observational	≥37	41	To find out the clinically useful parameters for the assessment of HIE using NIRS	TSNIR-3	n.r.	n.r.	rSO_2_ in quiet condition and rSO2, HbO_2_ and Hb during the inhalation of oxygen may be helpful for HIE infants. rSO2 for the healthy group increased rapidly, with the increase 7 ± 2.3%, compared to 3 ± 1.5% in HIE infants
Jain et al. (45)	Prospective observational	>36	21	To examine the value of CrSO_2_	INVOS	Midfrontal	48 h	Higher absolute CrSO_2_ values during TH correlates with subcortical injury on MRI and poor neurodevelopmental outcome
Kovacsova et al. (46)	Observational	>36	55	To investigate the SRS algorithm using a multi-distance broadband NIRS device to derive tissue saturation	Broadband NIRS	Frontal	14 h	A broadband NIRS multi-distance device can provide additional information to improve the robustness of the SRS estimation of cerebral tissue saturation
Lee et al. (47)	Prospective observational		64	To examine whether optimizing cerebral autoregulation is associated with decreased brain injury	INVOS 5100	Bilateral forehead	90 h	Blood pressure deviation from the optimal vasoreactivity was associated with evidence of brain injury on MRI, independent of initial birth asphyxia
Lemmers et al. (48)	Observational	≥36	39	To re-evaluate the early predictive value of rScO_2_, cFTOE and aEEG background pattern for outcome	INVOS 4100–5100, with adult sensor	Frontoparietal	84 h	Higher rScO_2_ values and lower aEEG background pattern scores in neonates with adverse neurodevelopmental outcome
Massaro et al. (49)	Prospective observational	>36	10	To assess cerebral perfusion and oxygenation differences after HIE	FORE-SIGHT	n.r.	84 h	Cerebral FTOE values were significantly reduced after rewarming in infants with evidence of injury on MR imaging
Massaro et al. (50)	Observational	≥35	36	To investigate if the duration and magnitude of the pressure passivity during TH were related to outcome	NIRO 200	Fronto-temporal	84 h	Higher PPI in both hemispheres and high gain on right hemisphere were associated with poor outcome
Meek et al. (2)	Observational	≥36	27	To measure changes in cerebral hemodynamics during the first 24 hrs of life after perinatal asphyxia, and relate them to outcome	NIRO1000 or NIRO500	n.r.	1–4 occasions between 2 and 72 h of age	increase in CBV on the 1st day of life is a sensitive predictor of adverse outcome. A reduction in CBVR is almost universally seen following asphyxia, but is not significantly correlated with severity of adverse outcome
Mitra et al. (51)	Prospective observational	≥35	14	To assess the cerebral metabolic and hemodynamic changes during the rewarming period after TH	Broadband NIRS	Frontal	14 h	The relationship between mitochondrial metabolism and oxygenation became impaired with rising Lac/NAA. Cardiovascular parameters remained stable during rewarming.
Mitra et al. (52)	Prospective observational	>34	23	To investigate the effects of disturbances in brain metabolism following HIE on outcome, using a wavelet based metabolic reactivity index between oxCCO and MABP	Broadband NIRS	Frontal	1 h	Pressure passive changes in brain metabolism were associated with injury severity and outcome following HIE. oxCCO-MABP semblance as a metabolic reactivity index correlated with MRS derived Lac/NAA. It also differed among groups of mild to moderate and severe injury based on MRI score and neuro-developmental outcome at 1 yr of age.
Mitra et al. (53)	Prospective observational	>36	14	To assess the changes in brain hemodynamics and metabolism following HIE in relation to initial degree on injury on EEG	Broadband NIRS	Frontal	12.5 h	Significant difference noted in derangement of brain oxygenation and metabolism between infants with mild and moderate to severe EEG abnormality
Nakamura et al. (54)	Observational	>35	11	To find the influence of CBV and ScO_2_ on clinical outcome	TRS-10	Parietal	72 h	Early postnatal CBV and ScO_2_ elevations were predictive of a poor outcome based on MRI injury
Niezen et al. (55)	Retrospective observational study	≥37	39	To determine the predictive value of aEEG and NIRS alone, and in combination, during the first 4 days after HIE	INVOS 5100C	left or right frontoparietal	96 h	After 48 h of TH, a higher rcSO_2_ was associated with a severely abnormal outcome
Peng et al. (56)	Observational	>=36	18	To assess whether NIRS Identifies the newborns during TH, who later develop brain injury	FORE-SIGHT	Forehead	79 h	rSO2 was consistently higher in newborns who developed brain injury on MRI and was significantly higher on day 1 compared to infants who did not develop injury on brain MRI.
Shellhaas et al. (57)	Observational	≥37	21	To evaluate the utility of aEEG and rSO_2_ for short-term outcome	INVOS 5100C	bilateral parietal regions, also one sensor over thigh	90 h	During day 3 of cooling and during rewarming, loss of physiologic variability (by systemic NIRS) was the best predictor of poor short-term outcome. Cerebral rSO2 variability was independent from short-term outcome
Shellhaas et al. (58)	Observational	≥35	4	To evaluate the variability of cerebral oxygen metabolism in sleep-wake states among sick neonates	INVOS 5100C	bilateral parietal-occipital regions	11.7 h	Cerebral oxygenation (sSO_2_) and FTOE significantly differ between wakefullness and sleep stages
Shellhaas et al. (59)	Observational	“term neonates”	18	To identify systemic and cerebral risk factors for adverse long-term neuro-developmental outcome following HIE	INVOS 5100C	Bilateral parietal regions, neonatal sensors	72 h	Mean cerebral rSO_2_ was not different between those with favorable vs. adverse 18-months outcomes, but those with favorable outcomes had higher systemic rSO_2_ variability during hours 48–72 of cooling
Tax et al. (60)	Observational	>34	38	To investigate peripheral oxygenation and perfusion in the first 48 h after perinatal asphyxia	NIRO 300	Left calf	n.r.	Peripheral oxygenation and perfusion are compromised with worsening degree of acidosis on cord blood gas
Tekes et al. (61)	Observational	≥35	27	To assess whether lower ADC values on MRI would correlate with worse autoregulatory status measured by NIRS	INVOS	Forehead bilateral	n.r.	Lower ADC scalars in the PCS, PLIC and PP correlated with blood pressure deviation below MAP_OPT_ during hypothermia and rewarming
Tian et al. (62)	Observational	≥36	9	Quantitative evaluation of cerebral autoregulation	INVOS 4100-5100, neonatal sensor	Frontoparietal	72 h	Cerebral autoregulation was time-scale –dependant. Both in phase and anti-phase coherence were related to worse outcome
Toet et al. (15)	Observational	>37	18	To determine the value of rSO_2_, FTOE measured by NIRS, and aEEG in relation to neuro-developmental outcome	INVOS 4100	Left parietal	48 h	rSO_2_ values remained normal and stable in infants with a normal outcome with values between 50 and 70% 30,33 but increased to supranormal values after 24 h in the infants with an adverse outcome. From 24 h onward, the values of rSO_2_ of the infants with an adverse outcome were significantly higher as compared with those with a favorable outcome
Van Bel et al. (18)	Observational	>35	31	To investigate whether cerebral perfusion and metabolism drops following hypoxia	Radiometer	Source on ant. fontanel, detector on Fronto-parietal	4–6 h	CBV, HbO, HbR, and Cytaa_3_ decreased in the first 12 hs of life in severely asphyxiated neonates who subsequently developed neurologic abnormalities
Wintermark et al. (63)	Observational	≥36	7	To determine the correlation between measurements of brain perfusion by NIRS and by MRI	FORE-SIGHT Cerebral Oximeter	Forehead	84 h	SctO_2_ and CBF increase from days 1 to 2 in all, despite TH. SctO_2_ and CBF are highly correlated in newborns with severe encephalopathy. Newborns with severe encephalopathy have lower CBF than newborns with moderate encephalopathy. Newborns developing brain HI injury have higher SctO_2_ than newborns not developing brain injury
Wu et al. (64)	Retrospective cohort study	≥36	20	To review the cerebral hemodynamic response during rewarming following TH	INVOS 5100C	Frontal region	14 h	CrSO_2_ and cerebral FTOE remained unchanged during rewarming
Zaramella et al. (65)	Case control study	≥36	22	To assess the diagnostic and prognostic value of TOI and ΔCBV in HIE	NIRO 300	Fronto-temporal	Duration n.r., study on day 1	Increased TOI on day 1 suggested abnormal outcome at 1 year of age

*ADC, Apparent diffusion coefficient; aEEG, Amplitude integrated electroencephalogram; HIE, Hypoxic-ischemic encephalopathy; CBO, Cerebral blood oxygenation; CBV, Cerebral blood volume; CCVR, Cerebral blood volume response; CrSO_2_, Cerebral regional oxygen saturation; Cytaa3, Cytochrome oxidase; DCS, Diffusion correlation spectroscopy; EEG, Electroencephalography; FDNIRS, Frequency-domain near-infrared spectroscopy; FTOE, Fractional tissue oxygen extraction; HbO, Oxy-hemoglobin; HbR, Deoxy-hemoglobin; MABP, Mean arterial blood pressure; MRI, Magnetic resonance imaging; MRS, Magnetic resonance spectroscopy; NE, Neonatal encephalopathy; NIRS, Near Infrared spectroscopy; NVC, Neurovascular coupling; oxCCO, Oxidation state of cytochrome c oxidase; PCS, Posterior centrum semiovale; PLIC, Posterior limb of internal capsule; PP, Putamen and globus pallidus; rCMRO_2_, Relative cerebral metabolic rate of oxygen consumption; rSO_2_, Regional oxygen saturation; rScO_2_, Regional cerebral tissue oxygen saturation; SctO_2_, Regional cerebral tissue oxygen saturation; StO_2_, Cerebral tissue oxygenation; TH, Therapeutic hypothermia; TOI, Tissue oxygenation index; TH, Therapeutic hypothermia; THI, Total hemoglobin index*.

## Discussion

Over the last decade, there has been increasing focus on NIRS monitoring at the cot side in babies with HIE and how it can inform clinical management and prognosis (Table 2).

## NIRS Devices and Methodology

Fifteen different NIRS instruments were used in the included studies, of which 11 instruments were commercially available. Among the non-commercial systems, a BNIRS system was used in eight studies (25–28, 46, 51–53), purpose-built to monitor concentration changes in oxCCO and hemoglobin parameters. A frequency-domain near-infrared spectroscopy (FD-NIRS) protocol in a customized commercial FD oximeter was used to measure cerebral oxygenation (35). This was also coupled with a diffuse correlation spectroscopy (DCS) system to measure an additional index of tissue CBF (41). Huang et al. used a prototype instrument (TSNIR-3, using SRS technology) to compare cerebral oxygenation between healthy controls and HIE infants (44).

Three different modes of NIRS have been developed and used in systems in clinical settings—continuous wave, time resolved, and frequency domain NIRS.

Continuous wave (CW) instruments were the earliest NIRS instruments to be developed and are the most commonly used. They use 2–4 discrete wavelength continuous sources [either laser or light-emitting diode (LED)] and measure the transmitted or reflected intensity through tissue. CW-NIRS systems are unable to separate attenuation of light due to absorption and scattering, so instead monitor changes in attenuation, and assume these are all due to absorption and that scattering remains stable within the measurement period. Using the modified Beer-Lambert law (66), these devices calculate changes in HbO_2_ and HHb concentration from an arbitrary baseline. Therefore, CW-NIRS protocols typically rely on variations in the signals or responses to specific (physiological or functional) stimuli.

In frequency domain NIRS (FD-NIRS) systems, the intensity of the emitted light is modulated at a particular frequency and the transmitted light attenuation and the frequency phase shift are measured. Observations of phase shift relate to tissue scattering, so FD-NIRS systems can derive absolute chromophore concentrations by decoupling absorption from scattering. This gives FD-NIRS a theoretical advantage of a more consistent quantitative measurement which can be important for cerebral NIRS.

The most complex NIRS mode, time domain NIRS (TD-NIRS) uses ultrashort pulses of light and measures the time of flight through the tissue with a photon counting device. This enables quantification of scattering and chromophore concentrations absolutely. An additional advantage of time resolved NIRS is the ability to gate the signal to obtain depth resolution. The disadvantages of this technology are the large instrument size and higher cost, although technological advances make up for these drawbacks (67). Both FD-NIRS and TD-NIRS systems appear to have superior depth sensitivity compared to CW-NIRS system (68).

The most common use of NIRS in the neonatal intensive care is a variation on CW-NIRS or FD-NIRS called cerebral oximetry, which allows the quantification of the oxygen saturation within tissue as a percentage using multi-distance approaches. These enable the recovery of scaled absolute measurements using a variety of different techniques (most commercial methods are not published). The NIRO series brain oximeter devices from Hamamatsu implements the spatially resolved spectroscopy (SRS) algorithm with their multi-distance measurements (69, 70). The multi-distance designs are less sensitive to changes in the extracerebral layers, giving more brain-specific measurements (71). Cerebral oxygenation represents the combined oxygen saturation of the arterial and venous vascular compartments, weighted by their volume (ratio of arterial and venous vessels in cerebral tissue is ~25:75%). The simplicity of the reading as an absolute number, small size and low weight of the instrument as well as the high sampling rate makes it ideal for bedside monitoring.

Most commercially available NIRS instruments only measure brain tissue light attenuation at 2–4 wavelengths to resolve hemoglobin oxygenation. However, broadband NIRS devices measure brain tissue light attenuation over a wide range of NIR wavelengths, allowing enhanced spectroscopic information and the possibility to resolve multiple chromophores. Broadband NIRS systems are particularly useful for monitoring changes in CCO (53) together with tissue saturation (46, 72). Due to the relatively low concentration of CCO *in vivo*, the selection of the specific wavelengths and number of wavelengths becomes an important factor for monitoring CCO. Broadband NIRS devices are able to accurately quantify changes in the oxidation state of CCO (oxCCO) by using over 100 wavelengths to improve the optical signal and extract the relatively low concentration changes (19).

DCS measures the microvascular blood flow in the biological tissue. It uses a NIR laser to measure temporal fluctuations in the detected laser intensity which are directly proportional to the speed of scatterers (mainly red blood cells) within the tissue. DCS has been used in combination with a multidistance FD-NIRS system to measure an index of the cerebral metabolic rate of oxygen (CMRO_2_) (35).

## Monitoring Cerebral Oxygenation

Thirty-five studies used cerebral oximetry measurements to describe changes in cerebral tissue oxygen saturation. Different oximetry terminologies have been described in Table 2. Seven studies have also used hemoglobin difference (HbD) as a marker of tissue oxygenation. Although cerebral oximetry presents an absolute value, HbD is measured as a change in concentration (27, 52).

Studies conducted continuously over the period of TH and rewarming revealed a difference in cerebral oxygenation between the groups of infants with good and poor outcome (56, 61). Cerebral oxygenation drops in the first 4–6 h of life following HI injury and recovers by 18–20 h (56). This post-HI drop in cerebral oxygenation was less evident in infants who subsequently develop brain injury (61). Peng et al. described a significant difference in cerebral oxygenation from birth till first 12 h of life between groups with evidence or absence of injury on MRI (56), while Lemmers et al. indicated a significant difference from 24 h onwards between groups of favorable and adverse neurodevelopmental outcomes at 2 years of age (48). The sensitivity and specificity of cerebral oxygenation to predict the adverse outcome within the first 10 h of TH were 100 and 83% in the study by Peng et al. (56). Lemmers et al. identified the highest predictive value at 24–30 h of life with a sensitivity of 92% and specificity of 64% (48). Higher cerebral oxygenation between 24 and 36 h of life also significantly increased the odds of having moderate to severe injury on MRI (45). This increased cerebral oxygenation is likely to be related to mitochondrial dysfunction or injury, reflected by decreased oxygen utilization. In addition, vasoparesis and luxury perfusion in the “secondary energy failure” stage, with cerebral perfusion exceeding the metabolic demand results in higher cerebral oxygenation in infants with severe injury.

Van Bel et al. reviewed cerebral hemodynamics and oxygenation responses in babies with HIE (18) in the pre-hypothermic era - CBV, HbO_2_, and HbR (deoxy-hemoglobin) decreased in the first 12 h of life in severely asphyxiated infants with the parameters becoming more stable between 12 and 24 h of life. Meek et al. also noted increased CBF and CBV on the 1st day of life in infants with severe HIE at presentation (Sarnat stage III) (2). These findings were further supported by the study from Nakamura et al. (54). CBV was significantly higher in the poor outcome group (three cooled and two non-cooled infants) at 6 h of age and by 24 h of age, cerebral oxygenation was significantly higher in the same group when compared to the infants with favorable outcome based on MRI findings. Cerebral oxygenation together with CBV at 24 h had a sensitivity, specificity, PPV and NPV of 100% for the predictive ability for neurological outcome based on MRI findings between 7 and 14 days after birth.

## Monitoring Mitochondrial Oxidative Metabolism

Jöbsis in his seminal paper in Science (17) reported a new optical method (NIRS) intended to monitor changes in concentration of cytochrome c oxidase along with changes in oxy- and deoxy- hemoglobin for use as a clinical tool. Using a commercial NIRS system (radiometer using four wavelengths—904, 845, 805, and 775 nm) van Bel et al. described a decrease in Cytaa_3_ (cytochrome oxidase) with increasing postnatal age in infants with severe HIE in the pre-TH era. Recently, the UCL group monitored Δ[oxCCO] in a preclinical model using a broadband NIRS (BNIRS) and described a significant correlation between the indicators of brain energy state on phosphorus (^31^P) MRS [phosphocreatine/exchangeable phosphate pool (PCr/epp) and total nucleotide triphosphate/exchangeable phosphate pool (NTP/epp)] and Δ[oxCCO] during and after HI insult (73). Bale et al. subsequently described a new BNIRS instrument for clinical research (CYRIL, using 136 wavelengths, 770–906 nm) (25) and investigated how the relationship between the changes in [oxCCO] and systemic physiology was associated with injury severity (26). Mitra et al. presented a metabolic reactivity index using wavelet analysis (wavelet semblance or phase relationship between two variables) between [oxCCO] and MABP at 48 h of life during TH following HIE that differentiated between infants with good and poor outcome (based on MRI scores, thalamic MRS outcome biomarker (Lactate/N-acetyl aspartate) and neurodevelopmental outcome) (52). The relationship between cerebral oxygen delivery and mitochondrial oxidative metabolism also indicated injury severity during TH (27) and rewarming (51, 53). Findings from these studies using broadband NIRS are consistent with known pathophysiological changes following HIE and indicate deranged oxidative metabolism resulting from mitochondrial injury and altered hemodynamics associated with the neurochemical cascade effects on cerebrovascular tone.

Fractional tissue oxygen extraction (FTOE, five studies) and cerebral metabolic rate of oxygen consumption (CMRO_2_, two studies) have also been used to monitor cerebral oxidative metabolism. FTOE decreased from 24 h of age in the adverse outcome group as compared with the favorable outcome group in two studies from the Utrecht group in the cooling and pre-cooling era (15, 48), indicating an inability to utilize available oxygen due to more severe mitochondrial injury. Using an FD-NIRS–DCS system, the Harvard group (35, 41) successfully monitored CBF and calculated CMRO_2_ using Fick's principle. Increased CMRO_2_ and CBV were more sensitive markers of evolving neuronal injury compared to brain tissue oxygenation in a cohort of infants with evidence of brain injury on ultrasound and MRI following HIE (3 cases) and other etiologies in the pre-cooling era (41). A subsequent study from the same group in a cohort of infants undergoing TH (10 infants with HIE and 17 matched control infants) (35) presented lower CMRO_2_ and CBF and high CBV in infants during TH compared to control infants and post-TH values. The reason for difference in findings of CMRO_2_ was not clear, but CMRO_2_ depends on CBF which was also low in the second study and was not recorded in the first study. In the first study, all three infants had a severe injury (intensive care withdrawn in two cases and the third case developed significant impairment), but in the second study, eight infants had a comparatively milder injury (either normal MRI or decreased apparent diffusion coefficient (ADC) in the cortex and white matter). The neurodevelopmental outcome in the second study at 18 months of age (56.6% normal outcome) was similar to those reported in the literature. The findings in the different neurodevelopmental groups were not explored in this study. Elevated CBV during TH was noted (consistent with previous studies) although CBF was decreased. Possible effects of medications (e.g., dopamine) and deranged cerebral autoregulation following HIE were discussed as possible factors contributing to these findings.

## Monitoring Cerebral Autoregulation

Cerebral autoregulation refers to the physiological ability of the healthy brain to maintain a steady cerebral blood flow (CBF) during changes in cerebral perfusion pressure (CPP). In view of the difficulty in direct measurement of invasive CPP, mean arterial blood pressure (MABP) is used as a proxy marker in neonatal studies. The relationship between cerebral oxygenation (as a marker of changes in CBF) or total hemoglobin concentration (as a marker of total blood volume) and MABP has been used to determine the autoregulatory capacity of the newborn brain both in time and frequency domain analysis. Cerebral vasoparesis following perinatal hypoxia ischaemia is associated with impaired pressure autoregulation leading to poor outcome (2). Failure to regulate CBF during changes in MABP following HIE can lead to the uncoupling of the tight relationship between CBF and cerebral energy metabolism and results in further injury during secondary energy failure.

Nine studies investigated changes in different cerebrovascular reactivity indices in relation to outcome. Massaro et al. (49) used spectral coherence that quantifies the relationship between changes in MABP and changes in HbD to identify pressure-passive cerebrovascular circulation indicated by increased coherence between the two physiological signals. Pressure passivity index (PPI) and gain were used for quantification of the duration and magnitude of cerebral pressure passivity, respectively. Infants with poor outcome (evidence of injury on MRI) exhibited higher PPI and gain, indicating longer duration and higher magnitude of cerebral pressure passivity following hypoxia-ischemia. Howlett et al. used a time domain-based reactivity index between hemoglobin volume and MABP (HVx) to identify an optimal blood pressure (MAP_OPT_) where vasoreactivity is greatest (43). A greater severity of brain injury was associated with more time spent with MABP below MAP_OPT_ during rewarming, while neonates with evidence of no or mild injury spent more time with MABP within or above MAP_OPT_. Burton et al. used the same index (HVx) and followed up 19 infants to 2 years of age (29). Infants with poor outcome at 2 years had higher MAP_OPT_ values, spent more time with MABP below MAP_OPT_ and had greater MABP deviation below MAP_OPT_ during rewarming. Also, infants with greater MABP deviation above MAP_OPT_ had lesser disability and higher cognitive scores. Lee et al. from the same group reviewed the role of HVx in a larger cohort of 64 infants and confirmed that greater duration and deviation of MABP below MAP_OPT_ were associated with greater injury in the white matter and paracentral gyri on MRI. MABP within MAP_OPT_ was associated with lesser injury in the white matter, putamen and globus pallidus, and brain stem (47). Restricted diffusion (characterized by low ADC values) in the posterior centrum semiovale and the posterior limb of the internal capsule correlated with MABP deviation below the MAP_OPT_ during hypothermia was also observed (61). Lower ADC scalars in the basal ganglia correlated with worse autoregulation during rewarming after TH.

Chalak et al. reviewed the dynamic and multiple-time-scale properties of cerebral autoregulation with a moving time window correlation between cerebral oxygenation and MABP and demonstrated the presence of large spontaneous fluctuations in MABP during TH in the infants with abnormal outcome (31). Both in-phase and antiphase correlations were associated with poor outcome. Tian et al. from the same group used wavelet analysis to understand and characterize the cerebrovascular reactivity in both time and frequency domain (62). The time-scale dependent nature of dynamic cerebral autoregulation was described with both in-phase and anti-phase coherence between the spontaneous oscillations in MABP and cerebral oxygenation. Findings were similar to their previous study (31). Mitra et al. presented a refined wavelet analysis technique and described a metabolic and haemodynamic reactivity index (wavelet semblance or phase difference between [oxCCO] and MABP or [HbD] and MABP]) in relation to outcome (52).

These findings highlight the importance of appropriate haemodynamic management following HIE for the prevention of secondary brain injury. This potential benefit of optimizing haemodynamic management using NIRS based reactivity indices requires validation.

## Cerebral NIRS Markers and Neurodevelopmental Outcome

Fifteen studies have included a neurodevelopmental follow up data to compare the NIRS based indices for outcome prognostication. Meek et al. followed up a cohort of infants following HIE to 1 year of age and noted a raised CBV in the adverse outcome group on day 1 (2). Toet et al. followed up their cohort up to 5 years of age using the Griffiths Mental Developmental Scale to identify the favorable and unfavorable groups in the pre-TH era (15). The same group has subsequently reviewed the prognostic value of cerebral oxygenation and FTOE in the cooling era in a cohort of 39 infants and noted significant differences in both cerebral oxygenation and FTOE between the good and adverse outcome groups (48). These findings have been discussed previously. Interestingly, in a study cohort of 18 infants (57), no definite relationship was noted by Shellhaas et al. between cerebral oxygenation and neurodevelopmental outcome at 18 months. Differences of these findings compared to previous studies can be related to: (a) smaller sample size; (b) decisions to withdraw intensive care were different; in the other two studies (15, 48) as most infants with a predicted adverse outcome died after redirection of care during the neonatal period; (c) use of different neurodevelopmental assessment tools—Shellhass et al. used Bayley Scales of Infant Development, Lemmers et al. and Toet et al. both used Griffiths Mental Developmental scales; and (d) use of different NIRS sensors—neonatal sensors were used by Shellhass et al. while the other studies used pediatric sensors. Different NIRS sensors are known to cause differences in absolute values of cerebral oxygenation (74, 75). Neonatal sensors tend to record tissue saturations higher than adult sensor (in case of INVOS NIRS monitors, this is ~10%). As most monitors have the upper limit set to 95%, higher values recorded by neonatal sensors can present in a straight line over time, without much variability.

Ancora et al. (23) noted a significantly higher cerebral oxygenation value at 12 h of age in infants with the adverse outcome on a 1-year Griffiths assessment. A trend toward higher values in the adverse outcome group was also observed at 6- and 24-h during TH. Similar findings were reported also by Zaramella et al. using the Amiel-Tison score at 1 year of age (65). Two recent studies (52, 62) using wavelet analysis also noted a clear difference in NIRS biomarkers between good and poor outcome infants based on their neurodevelopment assessment scores.

## NIRS Monitoring With Other Neuromonitoring Tools

Fifteen studies combined NIRS monitoring with structural and haemodynamic changes on MRI and metabolic derangement on thalamic ^1^H MRS while eight studies reviewed background electrical activity on aEEG together with NIRS.

Shellhas et al. (57), Peng et al. (56), and Mitra et al. (52) used different MRI scores to review the relationship between NIRS biomarkers with short term outcome. Massaro et al. (49) compared a measurement of CBF on Arterial spin labeling (ASL) MR imaging between 7 and 10 days with FTOE on day 1 (during cooling) and on day 4 (after rewarming) in infants with HIE and healthy controls. Infants with HIE had lower FTOE on both days (significantly lower after rewarming). Regional CBF on ASL in the basal ganglia thalamic (BGT) region and anterior white matter (AWM) was higher in the HIE cohort. However, CBF in BGT area in infants with no evidence of injury on MR imaging or watershed type of injury following HIE was higher in comparison to infants with confirmed evidence of injury in basal ganglia and focal/multifocal injury in the WM. The lack of hyperperfusion was thought to be related to the pseudonormalisation of CBF and low metabolic demand after the development of an irreversible injury. Wintermark et al. (63) further investigated the relationship between brain perfusion measured by NIRS and ASL-MRI. A strong correlation was noted between cerebral oxygenation and CBF measured with ASL MRI (mean CBF from both frontal lobes) in infants with severe HIE, although no significant correlation was found when both groups of infants with moderate and severe HIE were combined together. This study also demonstrated that infants with severe HIE had lower CBF and lower oxygen extraction compared to those with moderate HIE.

Tekes et al. reviewed the relationship between a NIRS marker of cerebrovascular reactivity (HVx) with diffusion weighted MR imaging (61). Blood pressure deviation from MABP_OPT_ (using HVx) was associated with low ADC scalers in the posterior limb of internal capsule (PLIC) and posterior centrum semiovale on MRI performed on day 10 of life or later. Howlett et al. used the same index to identify the optimal blood pressure that relates to outcome based on MRI findings (43). Mitra et al. used wavelet based NIRS reactivity indices to describe the relationship with proton (^1^H) MRS derived thalamic Lac/NAA (52).

The first study combining NIRS and aEEG monitoring was reported by Ancora et al. (22). A persistently abnormal aEEG at 24 h of life was not predictive of the adverse outcome but the recovery of electrical activity within this period was associated with good outcome (23). In comparison, high cerebral oxygenation at 12 h indicated poor neurodevelopmental outcome. In two Dutch studies (15, 48), higher cerebral oxygenation and lower aEEG background scores both were subsequently associated with poor outcome. Lemmers et al. were the first to evaluate a combined NIRS (cerebral oxygenation) and aEEG score for the prediction of neurodevelopmental outcome (48). The combined score had a significantly improved positive predictive value (91%) compared to individual monitoring (cerebral oxygenation 67%, aEEG 62%). This combined score helped to predict the outcome as early as 12 h of age (sensitivity 100%, specificity 87%). Improvement in predicting outcome using a combined score was also described by Goeral et al. (38) and Neizen et al. (55). Chalak et al. introduced an estimation of neurovascular coupling (NVC) using wavelet analysis of cerebral oxygenation and aEEG. A few examples were presented as case studies where this wavelet index of NVC was related to outcome (32).

Shellhaas et al. compared cerebral and somatic oxygenation on NIRS and aEEG with a composite score of short-term outcome (using Thompson scores on neurological examination after rewarming and MRI scores) (58). Absolute values of cerebral and somatic oxygenation, as well as the aEEG variables before and during rewarming did not correlate with short term outcome. However, the variability of systemic oxygenation was a good predictor of the short-term outcome, Variability of cerebral oxygenation was not related to outcome. The study presented only the analysis of data 6 h before rewarming and during the rewarming period (6 h), although their monitoring included the entire period of TH, rewarming period and 12 h of normothermia after completion of rewarming. It would have been useful to identify the trend of cerebral oxygenation on days 1–3 during TH in relation to this new short-term outcome composite score. The authors speculated that the multiorgan dysfunction resulting from HIE was reflected in low systemic oxygenation variability.

## Future Direction

Ideally, an optical neuromonitor in neonatal intensive care should provide continuous information regarding cerebral oxidative metabolism, oxygenation and blood flow in real-time at the cot side. Current studies using commercial NIRS oximeter systems can measure only cerebral oxygenation and in combination with other systemic measurements attempt to derive markers of metabolism. However, recent advances in NIRS technology and techniques have allowed the emergence of new directions in optical monitoring that promise to present a better insight into the degree of neural injury. New NIRS monitors that can monitor cerebral oxygenation and blood flow [Babylux: combining DCS and time-resolved reflectance spectroscopy (TRS) (76), Metaox: combining FD-NIRS and DCS (77)], Cyril: monitoring of CCO (BNIRS) and Florence: monitoring of CCO and blood flow together (combining BNIRS and DCS, currently being used by UCL group) are encouraging innovations in this area. There is continued research in the optical community to investigate the validity of existing tissue oximetry algorithms, especially regarding their precision and reproducibility (46, 78). New approaches, such as using broadband spectra (72) or novel combinations of spectral and multidistance techniques (79), are being developed to obtain more robust measurements of cerebral oxygenation. Advances in the optical developments to improve the accuracy of the measurement will increase acceptance within the clinical practice. The combination of (as opposed to the individual) measurements of brain tissue mitochondrial function, blood flow, oxygenation, and oxygen consumption will lead to a better assessment of neonatal hypoxic-ischaemic brain injury and most likely to offer enhanced prognostic value. An optical instrument that can deliver these measurements in real-time, non-invasively at the cot-side is necessary, with appropriate analysis techniques that will allow integration of these measurements toward the derivation of clinical information.

A small sample size often limits the validity of the findings of many NIRS studies. Future studies in this field need to be designed with sample size appropriate to answer the clinically relevant questions using measurable outcome parameters. This will provide further confidence in the clinical translation of this technology. Multicenter randomized controlled studies in the preterm population to review the benefit of NIRS monitoring have demonstrated the benefits of the cerebral oximetry monitoring (14), but no similar study has been published for HIE. One of the other complex issues is the use of different NIRS sensors and algorithms used by different manufacturers to measure cerebral oxygenation. Despite the difference in techniques and algorithms, most of the commercial monitors have shown a reasonable correlation between the measured tissue saturation values (74, 80–84) but without a uniform terminology, readers struggle to correlate the findings from different studies. Several manufacturers have developed smaller and flexible neonatal sensors but the use of different sensors with the same brain oximeter can produce different measurements, as pointed out by Lemmers et al. and colleagues (6, 74, 75). So, it is important to specify the type of NIRS oximeter sensor used with their reference values in each study. Finally, quantification of cerebral autoregulation using NIRS and systemic variables can offer a good insight of brain health; however, authors should consider the use of advanced signal processing techniques (e.g., wavelet analysis) to better quantify changes in the context of the dynamic nature of cerebral autoregulation.

## Limitations

We only reviewed human studies to focus on the assessment of this neuromonitoring technique in the clinical environment. This is a limitation of this review as some important work in preclinical models were not included. The use of the English language as a filter during the search and literature search using two medical databases might have resulted in the omission of some studies, although the chance of missing any major publication in this field will be low.

There are also some inherent limitations of NIRS technology. Any strong light (e.g., halogen spotlight attached to the incubator or a standing spotlight) can cause interference with NIRS monitoring. Hair can also sometimes pose an issue as it can absorb a lot of light, although this is unlikely in newborn infants. Hematoma and significant edema in the layers between skin and the scalp can also cause problems with NIRS recording as they will contribute to the measured signals, reducing the amount of information detected from the brain. Movement artifacts can also be an issue if not carefully documented.

## Conclusion

Significant effort has been made over the last decade to examine the role of cerebral NIRS monitoring in HIE. Commercially available cerebral NIRS parameters can identify cerebral hyperoxygenation, increased cerebral perfusion and loss of cerebral autoregulation in infants with severe HIE. Changes in NIRS variables in HIE are associated with subsequent neurodevelopmental outcome. Combined clinical neuromonitoring using NIRS and aEEG/EEG monitoring is feasible and appears to improve the prognostication of the neurodevelopmental outcome. Although the evidence from the currently available studies indicates a positive role for NIRS based neuromonitoring for infants with HIE, these findings need to be reviewed in larger prospective cohorts before translation to clinical practice.

## Author Contributions

SM completed the initial literature search. SM and NR assessed the papers and drafted the first review. SM, GB, JM, IT, and NR contributed to the final version.

## Conflict of Interest

The authors declare that the research was conducted in the absence of any commercial or financial relationships that could be construed as a potential conflict of interest.
